# Association between thyroid dysfunction and perinatal outcomes in women with gestational hypertension: a retrospective study

**DOI:** 10.1186/s12884-020-2805-6

**Published:** 2020-02-19

**Authors:** Juan Gui, Wangming Xu, Jie Zhang

**Affiliations:** 1 Dept. of Reproductive center, Renmin Hospital of Wuhan University, 238 Jiefang Road, Wuchang District, Wuhan, 430060 China; 2 Assisted Reproduction and Embryogenesis Clinical Research Center of Hubei Province, Wuhan, China

**Keywords:** Thyroid dysfunction, Hypothyroidism, Gestational hypertension, Severe preeclampsia, Early onset preeclampsia, Preterm birth

## Abstract

**Background:**

Previous studies showed that thyroid dysfunction in women with gestational hypertension could negatively affect maternal and fetal outcomes. In this study, we aimed to investigate whether thyroid dysfunction assessed in the second half trimester contributed to neonatal outcomes of pregnancy in different subtypes of gestational hypertension disease.

**Methods:**

We performed a retrospective case-control study and collected data from 135 singleton pregnant women with gestational hypertension disease and their offspring who delivered in Renmin Hospital of Wuhan University from January 2015 to June 2017. We classified the patients based on the severity of the preeclampsia into three groups: pregnant induced hypertension (PIH), mild preeclampsia (MPE) and severe preeclampsia (SPE). Based on the onset time of preeclampsia, we classified the patients into PIH, early onset preeclampsia (EPE) and late onset preeclampsia. Demographic data and levels of thyroid hormones, as well as the adverse maternal and neonatal outcomes were collected from Electronic Medical Records. Logistic regression was used to estimate the associations between thyroid dysfunction and neonatal outcomes in these patients.

**Results:**

Gestational weeks and neonatal birthweight were significantly lower, while incidence of preterm birth was significantly higher in the SPE and EPE groups than those in the PIH group (*P* < 0.001). Thyroid dysfunction was more frequent in the SPE group than in the MPE group (*P* = 0.01). Incidences of both preterm birth and low birth weight were significantly higher in patients with thyroid dysfunction (*P* = 0.008, *P* = 0.047 respectively). After adjustment, both severity of gestational hypertension (OR = 4.360, 95%CI [2.050, 9.271], *P* < 0.001; OR = 4.023, 95%CI [1.933, 8.372], *P* < 0.001) and thyroid dysfunction (OR = 3.011, 95%CI [1.248, 7.262], *P* = 0.014; OR = 11.306, 95%CI [1.040, 122.889], *P* = 0.046) were associated with higher risk of preterm birth and low birth weight, while the onset time of preeclampsia (OR = 0.031, 95%CI [0.009, 0.110], *P* < 0.001; OR = 0.097, 95%CI [0.033, 0.282], *P* < 0.001) was negatively associated with the risk of preterm birth and low birth weight.

**Conclusion:**

Severe and early onset preeclampsia, as well as thyroid dysfunction are associated with higher risk of preterm birth and low neonatal birth weight. Therefore, our data suggest that monitoring thyroid hormones in women with preeclampsia might help to predict adverse neonatal outcomes.

## Background

Preeclampsia is a major public health problem due to its frequency as well as its related maternal and perinatal morbidity and mortality, with a prevalence of 4.6% among pregnant women worldwide [1]. It is not only associated with adverse pregnant outcomes, but also contribute to higher risk of cardiovascular diseases, renal failure, type 2 diabetes mellitus, hypothyroidism and cognitive defects in future [2]. Furthermore, children born from preeclamptic pregnancies are more prone to hypertension, insulin resistance and diabetes mellitus, neurological complications, stroke, and mental disorders in their later lives [2].

Thyroid dysfunction is common among pregnant women. According to previous reports, the prevalence of clinical overt hyperthyroidism or subclinical hyperthyroidism is about 0.1–0.4% during pregnancy. The prevalence of hypothyroidism is about 2.5%, with clinical hypothyroidism accounting for 0.2–0.3%, and subclinical hypothyroidism for 2–3% [3, 4], while hypothyroxinemia for 1–2% [5]. According to domestic reports in China, the prevalence of gestational clinical hypothyroidism is approximately 0.5–0.6%, with subclinical hypothyroidism 2–3%, and hypothyroxinemia 1.6%. The prevalence of subclinical hypothyroidism is the highest.

Hypothyroidism is known to be one of the causes of hypertension [6]. Both untreated subclinical hypothyroidism and overt hypothyroidism are associated with multiple adverse outcomes in the mother and fetus [3, 7–23]. Women with treated hypothyroid diseases are not at higher risk than healthy pregnant women for adverse neonatal outcomes, but may also be at increased risk for preeclampsia [24]. However, Casey et al*.* do not find any increased incidence of preeclampsia in subclinical hypothyroid women [3]. Medici M et al. [25] have found that biochemical hyperthyroidism but not hypothyroidism during early pregnancy is associated with an increased risk of hypertensive disorders.

Therefore, the relationships between thyroid dysfunction and pregnancy outcomes in preeclampsia still needs to be clarified. It is known that the placental dysfunction is more likely to occur in the early onset preeclampsia at < 34 weeks of gestation [26, 27] and early onset preeclampsia conferred a substantially higher risk of cardiovascular [28], respiratory, central nervous system, renal, hepatic, and other morbidity [29]. However, in most previous studies, thyroid hormones were measured before 20 weeks of pregnancy. Associations of thyroid hormones measured in the second half of pregnancy in different subtypes of preeclampsia (mild and severe preeclamptic women as well as early onset and late onset women) and neonatal outcomes of pregnancy (rates of preterm birth and low neonatal birth weight) remained unclear and were investigated in the study.

## Patients and methods

### Study subjects

**Fig. 1:**
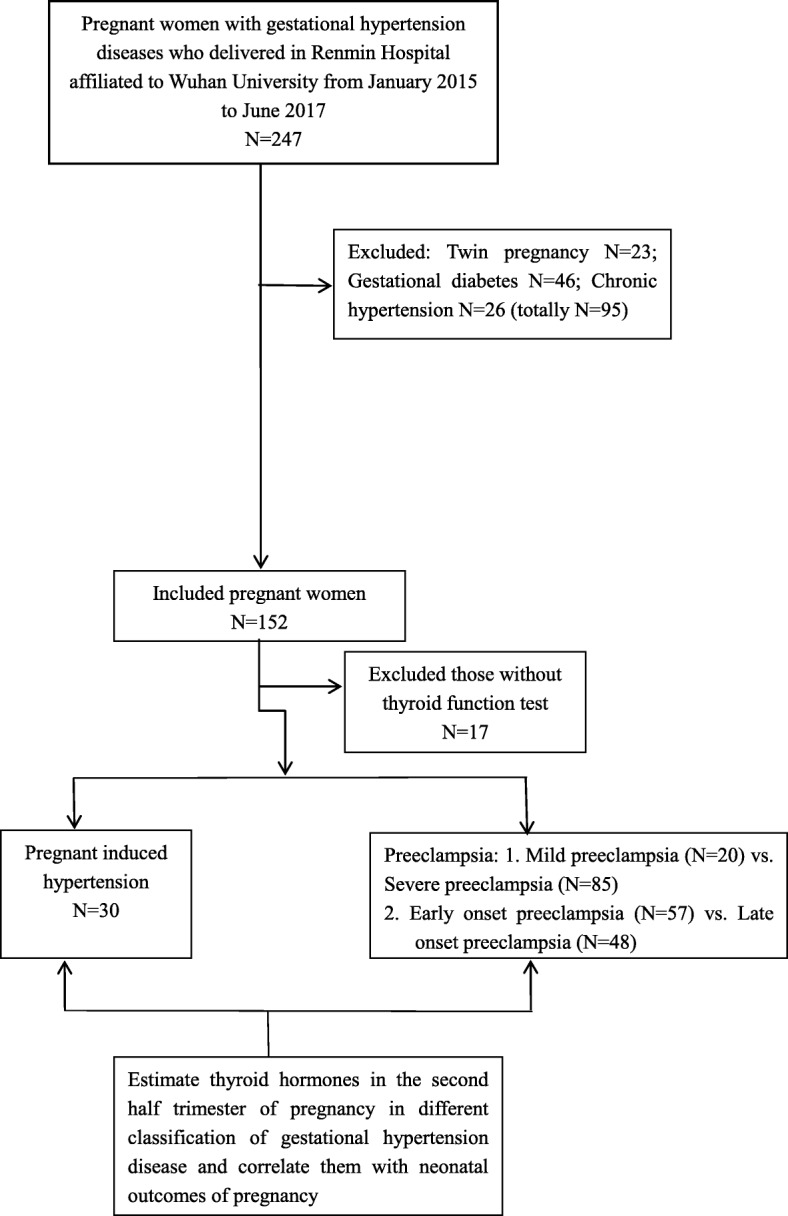
Flow chart and data processing

This was a retrospective case-control study. Overall, 135 singleton pregnant women who developed gestational hypertension disease and their offspring delivered in Renmin Hospital of Wuhan University from January 2015 to June 2017 were consecutively enrolled in the study. All the data were collected from the electronic medical record system, such as age, gestational age, prenatal examination, neonatal information and neonatal complications, etc. Blood routine, hepatic and renal functions, blood glucose, blood lipid, coagulation function and thyroid functions were all performed at the laboratory department of our hospital during hospitalization in the second half trimester of pregnancy. The primary outcomes were the maternal and fetal complications, i.e. preterm birth and low birth weight. The Ethics Committee of Renmin Hospital of Wuhan University approved the study (in accordance with the Helsinki declaration). Inclusion criteria: 1. maternal age between 20 and 40 years old; 2. no chronic diseases before pregnancy, such as chronic hypertension, cardiovascular and cerebrovascular diseases, autoimmune diseases (SLE etc.), thrombotic diseases, diabetes, thyroid endocrine diseases, hepatic and renal diseases, mental diseases, etc.; 3. it was a singleton pregnancy; 4. adequate iodine intake in daily diet; 5. delivered in our hospital. Exclusion criteria: 1. pregnant women with history of thyroid related diseases before pregnancy and taking medicines for thyroid diseases; 2. pregnant women with unhealthy diet habit; 3. pregnant women without follow-up; 4. pregnant women without results of thyroid function test; 5. pregnant women with diabetes or gestational diabetes or some other endocrine diseases. (Fig. 1. Flow chart and data processing).

### Definitions

Women without pre-existing hypertension were classified as having pregnant induced hypertension (PIH) if they had a systolic blood pressure ≥ 140 mmHg and/ or diastolic blood pressure ≥ 90 mmHg on at least two occasions first occurring after 20 gestational weeks.

Preeclampsia was defined as gestational hypertension in combination with one or more of the following new-onset conditions: proteinuria (urinary protein dip sticks ≥1+ or ≥ 300 mg/24-h); other maternal organ dysfunction, including renal insufficiency, hepatic dysfunction, neurological complications, and hematological complications; and uteroplacental dysfunction [30].

Preterm birth was defined if babies were born before 37 gestational weeks.

Low birth weight was defined as birth weight less than 2500 g.

According to the guidelines for the management of thyroid disorders during pregnancy and postpartum issued by the American thyroid association in 2017 [31], the definitions of thyroid diseases are as follows:

Clinical hypothyroidism: a. serum thyrotropic hormone (TSH) > 4 mIU/L, and serum free thyroxine (FT4) < lower limit of normal reference b. serum TSH > 10 mIU/L, with or without FT4 reduction.

Subclinical hypothyroidism: serum TSH > 4 mIU/L but no more than 10 mIU/L, serum FT4 is within the reference range.

Hypothyroxinemia: serum FT4 < the lower limit of normal reference value, and TSH is within the range of gestation-specific thyroid function reference value.

Gestational thyrotoxicosis: when TSH is less than 0.1 mIU/L, FT4 > gestational specific value reference upper limit.

Hashimoto thyroiditis: serum thyroid peroxidase antibody (TPOAb) ≥ 40 mIU/L.

Serum TSH, FT4, free triiodothyronine (FT3) and TPOAb levels were measured using electrochemi-luminescence immunoassay (Cobas Elesys 601, Roche Diagnostics). The reference ranges of FT4 in our hospital is 0.89–1.8 ng/dL; the reference ranges of FT3 in our hospital is 2.3–4.2 pg/mL.

### Groups

Based on the severity of the preeclampsia, we classified the patients into three groups 1) pregnancy induced hypertension (PIH), 2) mild preeclampsia (MPE), PIH with urinary protein dip sticks ≥1+ or ≥ 300 mg/24-h, and 3) severe preeclampsia (SPE), MPE plus at least one additional adverse condition including systolic blood pressure ≥ 160 mmHg and/ or diastolic blood pressure ≥ 110 mmHg or urinary protein dip sticks ≥3+ or ≥ 5 g/24-h or other maternal organ dysfunction. According to the onset time of the preeclampsia, we classified the patients also into three groups a) PIH b) early onset preeclampsia (EPE), preeclampsia appearing before 34 gestational weeks, and c) late onset preeclampsia (LPE), preeclampsia appearing at or after 34 gestational weeks [32]. The comparisons were among PIH, MPE and SPE, as well as among PIH, EPE and LPE.

### Statistical analysis

The data were expressed as frequencies or percentages for categorical variables and as means and standard deviations for normally distributed continuous variables, or medians and interquartile ranges for non-normally distributed continuous variables. Differences between the groups were compared by the chi-square or Fisher’s exact test for categorical variables and multiple comparisons in ANOVA or Kruskal Wallis Test for continuous variables. Associations between thyroid dysfunction, gestational hypertension disease and neonatal outcomes were assessed by using the logistic regression. Unadjusted and adjusted odds ratios (ORs, adjusted for age, gestational history, menstrual cycle, family history, history of gestational hypertension), as well as 95% confidence intervals (95%CIs) were calculated. *P* < 0.05 was considered to indicate statistical significance, while *P* < 0.017 was considered statistically significant for multiple comparisons in chi-square test and Kruskal Wallis Test. All calculations were performed using the SPSS Version 19.0.

## Results

### PIH, MPE, and SPE

Of the 135 patients, 30 were PIH, 20 were MPE, and 85 were SPE. There were 7 cases of fetal growth restriction (FGR), 3 cases of placental abruption and 2 cases of pleural effusion in these patients. All 7 cases of FGR, 2 cases of pleural effusion and 2 cases of placental abruption occurred in patients with SPE, while only one case of placental abruption was found in patients with MPE. Table 1 presents the clinical, biochemical markers and the incidences of adverse neonatal outcomes among PIH, MPE and SPE. One abortion, two induced labor and two fetal deaths were reported in the SPE group. The gestational week and neonatal birth weight were significantly lower, and the preterm birth rate was significantly higher in the SPE group than those in the other two groups (*P* < 0.001). Thyroid dysfunction was more frequent in the SPE group than in the MPE group (*P* = 0.01). Brain natriuretic peptide (BNP) was significantly higher in the SPE group than that in the PIH group (*P* = 0.002).

**Table 1:** Comparisons of the clinical data from three groups of gestational hypertension according to the severity of the disease

Characteristics	PIH (*N* = 30)	MPE (*N* = 20)	SPE (*N* = 85)	PIH vs. MPE	PIH vs. SPE	MPE vs. SPE
Age (year, mean ± SD)	30.43 ± 4.99	31.65 ± 4.44	31.45 ± 5.23	*P* = 0.407	*P* = 0.346	*P* = 0.876
Gestational weeks (at delivery) (week, mean ± SD)	38.53 ± 1.53	38.16 ± 2.37	34.42 ± 4.13	*P* = 0.899	*P* < 0.001	*P* < 0.001
Sex of baby (male, n%)	60.9% (14/23)	50% (8/16)	54.1% (40/74)	*P* = 0.730	*P* = 0.566	*P* = 0.768
Neonatal birth weight (g, mean ± SD)	3192.41 ± 629.84 (*N* = 29)	3134.21 ± 720.14 (*N* = 19)	2356.06 ± 787.95 (*N* = 71)	*P* = 0.791	*P* < 0.001	*P* < 0.001
Hematocrit (L/L, mean ± SD)	0.38 ± 0.03	0.37 ± 0.02	0.37 ± 0.04	*P* = 0.853	*P* = 0.328	*P* = 0.534
Hemoglobin (g/L, mean ± SD)	123.40 ± 9.36	123.90 ± 8.43	121.63 ± 14.41	*P* = 0.892	*P* = 0.514	*P* = 0.474
Platelet (10^9/L, mean ± SD)	220.53 ± 40.22	204.90 ± 45.86	194.38 ± 67.10	*P* = 0.524	*P* = 0.047	*P* = 0.797
Fibrinogen (g/L, mean ± SD)	4.06 ± 0.74	3.93 ± 0.63	3.73 ± 0.81	*P* = 0.575	*P* = 0.052	*P* = 0.301
D-Dimer (mg/L, medians and interquartile ranges)	1.67 (1.16–2.82)	1.76 (1.07–2.97)	1.84 (1.20–2.41)	*P* = 0.823	*P* = 0.607	*P* = 0.877
Brain natriuretic peptide (pg/mL, medians and interquartile ranges)	26.50 (17.25–65.50)	40 (21–90)	84.5 (34.75–231.25)	*P* = 0.265	*P* = 0.002	*P* = 0.075
FT3 (pg/mL, medians and interquartile ranges)	2.54 (2.24–2.69)	2.65 (2.52–2.89)	2.49 (2.26–2.70)	*P* = 0.040	*P* = 0.767	*P* = 0.014
FT4 (ng/dL, medians and interquartile ranges)	0.98 (0.90–1.06)	0.98 (0.86–1.06)	0.93 (0.85–1.06)	*P* = 0.992	*P* = 0.286	*P* = 0.311
TSH (mIU/L, medians and interquartile ranges)	2.32 (1.79–3.26)	2.09 (1.65–2.96)	2.58 (1.76–3.74)	*P* = 0.342	*P* = 0.881	*P* = 0.361
Triglyceride (mmol/L, medians and interquartile ranges)	3.56 (2.78–5.93)	4.15 (2.83–4.80)	3.98 (2.85–4.94)	*P* = 1.000	*P* = 0.974	*P* = 0.947
Total cholesterol (mmol/L, mean ± SD)	6.23 ± 1.06	5.25 ± 1.69	6.28 ± 1.75	*P* = 0.150	*P* = 0.998	*P* = 0.121
Low density lipoprotein (mmol/L, mean ± SD)	2.92 ± 0.93	2.15 ± 1.08	2.72 ± 1.39	*P* = 0.064	*P* = 0.490	*P* = 0.114
High density lipoprotein (mmol/L, mean ± SD)	1.95 ± 0.38	1.59 ± 0.73	1.76 ± 0.80	*P* = 0.126	*P* = 0.241	*P* = 0.408
Rate of preterm birth (n%)	7.1% (2/28)	15.8% (3/19)	58.6% (41/70)	*P* = 0.644	*P* < 0.001	*P* = 0.001
Rate of abortion (n%)	0	0	1.3% (1/75)	NS	NS	NS
Rate of induced labor (n%)	0	0	2.7% (2/75)	NS	NS	NS
Rate of fetal death (n%)	0	0	2.7% (2/75)	NS	NS	NS
Thyroid dysfunction (n%)	60% (18/30)	40% (8/20)	70.6% (60/85)	*P* = 0.166	*P* = 0.286	*P* = 0.010
Hypothyroidism (n%)	0	0	9.4% (8/85)	NS	NS	NS
Subclinical hypothyroidism (n%)	20% (6/30)	15% (3/20)	11.8% (10/85)	*P* = 0.748	*P* = 0.622	*P* = 0.988
FT3 under the lower limit (n%)	6.7% (2/30)	0	8.2% (7/85)	NS	*P* = 0.847	NS
FT4 under the lower limit (n%)	16.7% (5/30)	20% (4/20)	22.4% (19/85)	*P* = 1.000	*P* = 0.324	*P* = 0.330
FT3 and FT4 both under the lower limit (n%)	6.7% (2/30)	5% (1/20)	9.4% (8/85)	*P* = 1.000	*P* = 0.709	*P* = 0.517
Hashimoto thyroiditis (n%)	13.3% (4/30)	10% (2/20)	5.9% (5/85)	*P* = 0.66	*P* = 0.69	*P* = 1
hyperthyroidism (n%)	0	0	3.5% (3/85)	NS	NS	NS

A nominal 2-sided probability value < 0.05 was considered to indicate statistical significance, and adjustments were made for multiple comparisons in chi-square test and Kruskal Wallis Test (*P*_adjust_ < 0.017)

*PIH* pregnant induced hypertension; *MPE* mild preeclampsia; *SPE* severe preeclampsia; *FT3* free triiodothyronine; *FT4* free thyroxine; *TSH* thyrotropic hormone

### PIH, EPE, and LPE

Table 2 shows the clinical, biochemical markers and the incidences of adverse neonatal outcomes among the PIH, EPE and LPE groups. There were 57 EPE and 48 LPE. One abortion and two induced labor were reported in the EPE group. Both EPE and LPE groups had one fetal death. There were 4 cases of FGR, 2 cases of placenta abruption and 2 cases of pleural effusion in the EPE group, 3 cases of FGR and one case of placenta abruption in the LPE group. BNP was significantly higher in the EPE group and in the LPE group than in the PIH group (*P* = 0.010, *P* = 0.005 respectively). The gestational week was significantly earlier, the neonatal birth weight was significantly lower, and the preterm birth rate was significantly higher in the EPE group compared with the other two groups (*P* < 0.001). The EPE group also had the highest rate of thyroid dysfunction (71.9%).

**Table 2:** Comparisons of the clinical data from three groups of gestational hypertension according to the gestational age

Characteristics	PIH (*N* = 30)	EPE (*N* = 57)	LPE (*N* = 48)	PIH vs. EPE	PIH vs. LPE	EPE vs. LPE
Age (year, mean ± SD)	30.43 ± 4.99	31.61 ± 5.34	31.34 ± 4.78	*P* = 0.304	*P* = 0.445	*P* = 0.785
Gestational weeks (at delivery) (week, mean ± SD)	38.53 ± 1.53	32.70 ± 3.95	38.01 ± 1.82	*P* < 0.001	*P* = 0.454	*P* < 0.001
Sex of baby (male, n%)	60.9% (14/23)	48.9% (23/47)	58.1% (25/43)	*P* = 0.348	*P* = 0.830	*P* = 0.382
Neonatal birth weight (g, mean ± SD)	3192.41 ± 629.84 (*N* = 29)	1989.09 ± 712.97 (*N* = 44)	3028.48 ± 591.00 (*N* = 46)	*P* < 0.001	*P* = 0.288	*P* < 0.001
Hematocrit (L/L, mean ± SD)	0.38 ± 0.03	0.37 ± 0.03	0.37 ± 0.04	*P* = 0.795	*P* = 0.164	*P* = 0.175
Hemoglobin (g/L, mean ± SD)	123.40 ± 9.36	123.91 ± 12.03	119.83 ± 14.84	*P* = 0.857	*P* = 0.227	*P* = 0.102
Platelet (10^9/L, mean ± SD)	220.53 ± 40.22	208.10 ± 58.23	182.95 ± 66.72	*P* = 0.588	*P* = 0.011	*P* = 0.154
Fibrinogen (g/L, mean ± SD)	4.06 ± 0.74	3.67 ± 0.88	3.88 ± 0.64	*P* = 0.031	*P* = 0.338	*P* = 0.169
D-Dimer (mg/L, medians and interquartile ranges)	1.67 (1.16–2.82)	1.81 (1.09–2.39)	1.87 (1.27–2.66)	*P* = 0.390	*P* = 0.966	*P* = 0.371
Brain natriuretic peptide (pg/mL, medians and interquartile ranges)	26.50 (17.25–65.50)	78.50 (25.75–228.00)	84 (32–186)	*P* = 0.010	*P* = 0.005	*P* = 0.993
FT3 (pg/mL, medians and interquartile ranges)	3.56 (2.78–5.93)	3.48 (2.82–4.62)	4.32 (3.10–5.37)	*P* = 0.659	*P* = 0.621	*P* = 0.105
FT4 (ng/dL, medians and interquartile ranges)	6.23 ± 1.06	6.21 ± 1.69	6.00 ± 1.89	*P* = 1.000	*P* = 0.885	*P* = 0.925
TSH (mIU/L, medians and interquartile ranges)	2.92 ± 0.93	2.64 ± 1.43	2.62 ± 1.26	*P* = 0.366	*P* = 0.347	*P* = 0.923
Triglyceride (mmol/L, medians and interquartile ranges)	1.95 ± 0.38	1.71 ± 0.82	1.77 ± 0.75	*P* = 0.165	*P* = 0.300	*P* = 0.730
Total cholesterol (mmol/L, mean ± SD)	2.54 (2.24–2.68)	2.49 (2.26–2.76)	2.55 (2.35–2.70)	*P* = 0.989	*P* = 0.569	*P* = 0.518
Low density lipoprotein (mmol/L, mean ± SD)	0.98 (0.90–1.06)	0.95 (0.85–1.06)	0.93 (0.83–1.05)	*P* = 0.655	*P* = 0.229	*P* = 0.477
High density lipoprotein (mmol/L, mean ± SD)	2.32 (1.79–3.26)	2.61 (1.63–4.20)	2.18 (1.67–3.05)	*P* = 0.748	*P* = 0.534	*P* = 0.433
Rate of preterm birth (n%)	7.1% (2/28)	84.1% (37/44)	15.6% (7/45)	*P* < 0.001	*P* = 0.486	*P* < 0.001
Rate of abortion (n%)	0	2.1% (1/48)	0	NS	NS	NS
Rate of induced labor (n%)	0	4.2% (2/48)	0	NS	NS	NS
Rate of fetal death (n%)	0	2.1% (1/48)	2.2% (1/46)	NS	NS	*P* = 0.315
Thyroid dysfunction (n%)	60% (18/30)	71.9% (41/57)	56.3% (27/48)	*P* = 0.258	*P* = 0.744	*P* = 0.094
Hypothyroidism (n%)	0	10.5% (6/57)	4.2% (2/48)	NS	NS	*P* = 0.162
Subclinical hypothyroidism (n%)	20% (6/30)	15.8% (9/57)	8.3% (4/48)	*P* = 0.804	*P* = 0.284	*P* = 0.072
FT3 under the lower limit (n%)	6.7% (2/30)	10.5% (6/57)	2.1% (1/48)	*P* = 0.514	*P* = 0.680	*P* = 0.072
FT4 under the lower limit (n%)	16.7% (5/30)	19.3% (11/57)	25% (12/48)	*P* = 0.339	*P* = 0.623	*P* = 0.556
FT3 and FT4 both under the lower limit (n%)	6.7% (2/30)	7.0% (4/57)	10.4% (5/48)	*P* = 0.909	*P* = 1.000	*P* = 1.000
Hashimoto thyroiditis (n%)	13.3% (4/30)	8.8% (5/57)	4.2% (2/48)	*P* = 1.000	*P* = 0.303	*P* = 0.267
hyperthyroidism (n%)	0	3.5% (2/57)	2.1% (1/48)	NS	NS	*P* = 0.773

A nominal 2-sided probability value < 0.05 was considered to indicate statistical significance, and adjustments were made for multiple comparisons in chi-square test and Kruskal Wallis Test (*P*_adjust_ < 0.017)

*PIH* pregnant induced hypertension; *EPE* early onset preeclampsia; *LPE* late onset preeclampsia; *FT3* free triiodothyronine; *FT4* free thyroxine; *TSH* thyrotropic hormone

### Associations between gestational hypertension disease, thyroid dysfunction and neonatal outcomes

The rates of preterm birth and low birth weight was significantly higher in the patients with thyroid dysfunction than those without (49.3% vs. 25%, *P* = 0.008; 45.1% vs. 27.1%, *P* = 0.047). In the study population, both the severity of gestational hypertension (OR = 4.360, 95%CI [2.050, 9.271], *P* < 0.001) and thyroid dysfunction (OR = 3.011, 95%CI [1.248, 7.262], *P* = 0.014) were associated with a higher risk of preterm birth, while the onset time of preeclampsia (OR = 0.031, 95%CI [0.009, 0.110], *P* < 0.001) was negatively associated with the risk of preterm birth. Similarly, both the severity of gestational hypertension (OR = 4.023, 95%CI [1.933, 8.372], *P* < 0.001) and hypothyroidism (OR = 11.306, 95%CI [1.040, 122.889], *P* = 0.046) were associated with an increased risk of low birth weight, while the onset time of preeclampsia (OR = 0.097, 95%CI [0.033, 0.282], *P* < 0.001) was negatively associated with the risk of low birth weight. Results of logistic regression analysis for thyroid dysfunction and fetal outcomes were summarized in Table 3.

**Table 3:** Logistic regression results for the associations between gestational hypertension disease, thyroid dysfunction and neonatal outcomes (preterm birth and low birth weight)

Characteristics	preterm birth		low birth weight	
	Unadjusted	Adjusted	Unadjusted	Adjusted
severity of gestational hypertension (PIH, MPE, SPE)				
OR	4.914	4.360	4.119	4.023
95% CI	2.354–10.255	2.050–9.271	2.084–8.144	1.933–8.372
*P* Value	< 0.001	< 0.001	< 0.001	< 0.001
Onset time of gestational hypertension (EPE, LPE)				
OR	0.036	0.331	0.1108	0.097
95% CI	0.011–0.112	0.009–0.110	0.041–0.283	0.033–0.282
P Value	< 0.001	< 0.001	< 0.001	< 0.001
Thyroid dysfunction				
OR	2.829	3.011	2.140	2.187
95% CI	1.261–6.346	1.248–7.262	0.969–4.726	0.919–5.208
P Value	0.012	0.014	0.060	0.077
Hypothyroidism				
OR	6.182	3.373	15.455	11.306
95% CI	0.993–38.478	0.411–33.893	1.625–146.973	1.040–122.889
P Value	0.051	0.242	0.017	0.046
Both FT3 and FT4 under the lower limit				
OR	1.448	1.496	1.493	1.531
95% CI	1.064–1.971	1.000–2.237	1.103–2.021	1.049–2.234
P Value	0.019	0.242	0.009	0.027

Adjusted for age, gestational history, menstrual cycle, family history, and history of gestational hypertension

*PIH* pregnant induced hypertension; *MPE* mild preeclampsia; *SPE* severe preeclampsia; *EPE* early onset preeclampsia; *LPE* late onset preeclampsia; *FT3* free triiodothyronine; *FT4* free thyroxine; *TSH* thyrotropic hormone

## Discussion

In this retrospective case-control study, we found that patients with severe preeclampsia, early onset preeclampsia or thyroid dysfunction had higher risk of adverse maternal and fetal outcomes such as preterm birth and low neonatal birth weight. Our data show that the rates of preterm birth and thyroid dysfunction were the highest in the patients with EPE. Although we could not find significant differences between EPE and PIH groups in the rates of thyroid dysfunction (71.9% vs. 56.7%), giving more emphasis on the thyroid hormones in those patients with EPE might reduce some adverse maternal and fetal outcomes.

Preeclampsia is one of the major causes of maternal and perinatal death [33]. To better manage this disease, we need to improve our knowledge to better identify patients with preeclampsia at increased risk for adverse outcomes. In this study, most maternal adverse outcomes happened in patients with SPE or EPE. We found that BNP was significantly higher in these two groups which was in accordance with the symptoms. However, we did not find any difference among these groups in blood lipids. Most previous studies have found that thyroid dysfunction occurs more frequently in women with preeclampsia than in normal pregnant women [8, 18], and is associated with many adverse pregnant outcomes such as spontaneous abortion, intrauterine fetal death, preterm birth, and low birth weight etc. [3, 7, 11–13, 16, 17, 34] Our results are in accordance with these previous studies.

Hypothyroidism has been shown to have various vascular pathogenic effects, including endothelial cell dysfunction [35] which is also a pathophysiological basis of gestational hypertension. A study including 16,364 singleton births hypothyroid mothers in Finland found that maternal hypothyroidism was associated with higher risks of gestational hypertension (OR = 1.20, 95% CI [1.10–1.30]), severe preeclampsia (OR = 1.38, 95% CI [1.15–1.65]), preterm births (OR = 1.25, 95% CI [1.16–1.34]), major congenital anomalies (OR = 1.14, 95%CI [1.06–1.22]), and neonatal intensive care unit admission (OR = 1.23, 95% CI [1.17–1.29]) [36]. Surks et al. [37] showed that increased maternal serum TSH (higher than 10 mIU/L) was associated with increased risk of stillbirth. A study including 25,756 women conducted by Casey and colleagues [3] revealed that subclinical hypothyroidism in pregnancies was associated with a 3-fold increased risk of placental abruption (relative risk 3.0, 95% CI [1.1–8.2]). The risk of preterm birth was almost 2-fold higher in women with subclinical hypothyroidism than in those without (relative risk, 1.8, 95% CI [1.1–2.9]). Another study showed that the incidence of premature birth, low birth weight and neonatal asphyxia was significantly higher in pregnant women with hyperthyroidism than that in normal pregnant women [38]. In our study, only two types of thyroid dysfunction - i.e. hypothyroidism and FT3 and FT4 both below the normal lower limit - were found to be significantly associated with increased risk of low neonatal birth weight. The rate of hyperthyroidism was rare in this population. It might be due to the small sample size of our study or the racial differences of different studies.

Many studies focused only on the first trimester [9, 19, 25, 39, 40], and did not include all preeclampsia types [41]. The present study investigated thyroid hormones of women with gestational hypertension in the second half trimester and compared various types of preeclampsia according to the severity and the gestational age. Our results were also in consistency with other studies in the second half trimester. A study including 6031 mothers showed that after normalization of the thyroid hormones with appropriate treatment in women developing hypothyroidism in the first trimester, there was no significant difference in the risk of developing preeclampsia compared with the normal pregnant women. However, if the women developed hypothyroidism in the third trimester, they still had a 2.18-fold higher risk of developing preeclampsia [34]. A prospective study in China [42] including 3398 pregnant women found that isolated maternal hypothyroxinemia (IMH) in the first trimester did not increase the risk of adverse outcomes irrespective of whether women received L-thyroxine treatment or not. However, IMH identified in the second trimester was associated with a significantly increased risk of adverse pregnancy outcomes. The results suggest that thyroid function follow-up during the second trimester is necessary, even if thyroid function is normal during the first trimester. Therefore, comprehensive monitoring of thyroid hormones throughout the whole pregnancy and early treatment are very important to reduce the risk of preterm birth and low birth weight. Consequently, the rate of neonatal intensive care unit will be decreased and the burden to the family and the society will also be reduced. Thus, it might be helpful to test thyroid hormones in women with preeclampsia in all three stages, treatment should be initiated as early as possible by thyroid dysfunction to avoid adverse pregnant outcomes.

Our study had a few limitations. First, as a retrospective investigation, we could only analyze variables that were appropriately documented, and we were not able to investigate those patients without results of thyroid hormones. Second, this was a single center study with a small sample size, so the sensitivity and specificity of results were not quite satisfactory. With the popularity of thyroid function tests, we will get more accurate and precise data in China. Due to the retrospective design and the small size of our study, currently we cannot determine whether thyroid dysfunction in the first half trimester or in the second half trimester have a greater effect on maternal and fetal pregnancy outcomes. Data are also lacking regarding whether the treatment is obligatory for patients with FT3 and FT4 both under the lower limit but normal TSH to prevent adverse neonatal outcomes. Certainly, further studies are needed to widen our understanding on thyroid dysfunction and neonatal outcomes.

## Conclusions

Our data indicate that more severe and early onset preeclampsia, as well as thyroid dysfunction were associated with significantly increased risk of neonatal adverse outcomes such as preterm birth and low neonatal birth weight. Therefore, monitoring thyroid hormones in women with preeclampsia might help to predict and consequently reduce the adverse neonatal outcomes.

## Data Availability

The data that support the findings of this study are available from Electronic medical records of Renmin hospital of Wuhan University but restrictions apply to the availability of these data, which were used under license for the current study, and so are not publicly available. Data are however available from the authors upon reasonable request and with permission of Renmin hospital of Wuhan University.

## Abbreviations

BNP: Brain natriuretic peptide
EPE: Early onset preeclampsia
FGR: Fetal growth restriction
FT3: Free triiodothyronine
FT4: Serum free thyroxine
IMH: Isolated maternal hypothyroxinemia
LPE: Late onset preeclampsia
MPE: Mild preeclampsia
PIH: Pregnant induced hypertension
SPE: Severe preeclampsia
TPOAb: Serum thyroid peroxidase antibody
TSH: Serum thyrotropic hormone

## References

[1] Abalos E, Cuesta C, Grosso AL, Chou D, Say L (2013). Global and regional estimates of preeclampsia and eclampsia: a systematic review. Eur J Obstet Gynecol Reprod Biol.

[2] Tranquilli AL (2014). Prediction, medical illness and the risk of pre-eclampsia. Pregnancy Hypertens.

[3] Casey BM, Dashe JS, Wells CE (2005). Subclinical hypothyroidism and pregnancy outcomes. Obstet Gynecol.

[4] Cleary-Goldman J, Malone FD, Lambert-Messerlian G (2008). Maternal thyroid hypofunction and pregnancy outcome. Obstet Gynecol.

[5] Sahay RK, Nagesh VS (2012). Hypothyroidism in pregnancy. Indian J Endocrinol Metab.

[6] Endo T, Komiya I, Tsukui T (1979). Re-evaluation of a possible high incidence of hypertension in hypothyroid patients. Am Heart J.

[7] Feki M, Omar S, Menif O (2008). Thyroid disorders in pregnancy: frequency and association with selected diseases and obstetrical complications in Tunisian women. Clin Biochem.

[8] Allan WC, Haddow JE, Palomaki GE (2000). Maternal thyroid deficiency and pregnancy complications: implications for population screening. J Med Screen.

[9] Karakosta P, Alegakis D, Georgiou V (2012). Thyroid dysfunction and autoantibodies in early pregnancy are associated with increased risk of gestational diabetes and adverse birth outcomes. J Clin Endocrinol Metab.

[10] Schneuer FJ, Nassar N, Tasevski V, Morris JM, Roberts CL (2012). Association and predictive accuracy of high TSH serum levels in first trimester and adverse pregnancy outcomes. J Clin Endocrinol Metab.

[11] El Baba KA, Azar ST (2012). Thyroid dysfunction in pregnancy. Int J Gen Med.

[12] Sahu MT, Das V, Mittal S, Agarwal A, Sahu M (2010). Overt and subclinical thyroid dysfunction among Indian pregnant women and its effect on maternal and fetal outcome. Arch Gynecol Obstet.

[13] Hirsch D, Levy S, Nadler V, Kopel V, Shainberg B, Toledano Y (2013). Pregnancy outcomes in women with severe hypothyroidism. Eur J Endocrinol.

[14] Kumru P, Erdogdu E, Arisoy R (2015). Effect of thyroid dysfunction and autoimmunity on pregnancy outcomes in low risk population. Arch Gynecol Obstet.

[15] Zhou J, Li W, Du J, Qiao C, Shang T, Liu X (2014). Correlation between thyroid hormones and renal function in severe pre-eclampsia patients with hypothyroidism. Zhonghua Fu Chan Ke Za Zhi.

[16] Shinohara DR, Santos TDS, de Carvalho HC (2018). Pregnancy complications associated with maternal hypothyroidism: a systematic review. Obstet Gynecol Surv.

[17] Chen LM, Du WJ, Dai J (2014). Effects of subclinical hypothyroidism on maternal and perinatal outcomes during pregnancy: a single-center cohort study of a Chinese population. PLoS One.

[18] Wilson KL, Casey BM, McIntire DD, Halvorson LM, Cunningham FG (2012). Subclinical thyroid disease and the incidence of hypertension in pregnancy. Obstet Gynecol.

[19] Ashoor G, Maiz N, Rotas M, Kametas NA, Nicolaides KH (2010). Maternal thyroid function at 11 to 13 weeks of gestation and subsequent development of preeclampsia. Prenat Diagn.

[20] Negro R, Stagnaro-Green A (2014). Diagnosis and management of subclinical hypothyroidism in pregnancy. BMJ.

[21] Zhou J, Du J, Ma B (2014). Thyroid hormone changes in women with pre-eclampsia and its relationship with the presence of pre-eclampsia. Zhonghua Fu Chan Ke Za Zhi..

[22] Liu H, Shan Z, Li C (2014). Maternal subclinical hypothyroidism, thyroid autoimmunity, and the risk of miscarriage: a prospective cohort study. Thyroid.

[23] Tudela CM, Casey BM, McIntire DD, Cunningham FG (2012). Relationship of subclinical thyroid disease to the incidence of gestational diabetes. Obstet Gynecol.

[24] Wolfberg AJ, Lee-Parritz A, Peller AJ, Lieberman ES (2005). Obstetric and neonatal outcomes associated with maternal hypothyroid disease. J Matern Fetal Neonatal Med.

[25] Medici M, Korevaar TI, Schalekamp-Timmermans S (2014). Maternal early-pregnancy thyroid function is associated with subsequent hypertensive disorders of pregnancy: the generation R study. J Clin Endocrinol Metab.

[26] Li XL, Guo PL, Xue Y, Gou WL, Tong M, Chen Q (2016). An analysis of the differences between early and late preeclampsia with severe hypertension. Pregnancy Hypertens.

[27] Desforges Jane F., Cunningham F. Gary, Lindheimer Marshall D. (1992). Hypertension in Pregnancy. New England Journal of Medicine.

[28] Veerbeek JH, Hermes W, Breimer AY (2015). Cardiovascular disease risk factors after early-onset preeclampsia, late-onset preeclampsia, and pregnancy-induced hypertension. Hypertension.

[29] Lisonkova S, Sabr Y, Mayer C, Young C, Skoll A, Joseph KS (2014). Maternal morbidity associated with early-onset and late-onset preeclampsia. Obstet Gynecol.

[30] Hypertension in pregnancy. Report of the American College of Obstetricians and Gynecologists' Task Force on Hypertension in Pregnancy. Obstet Gynecol. 2013;122(5):1122–31. 10.1097/01.AOG.0000437382.03963.88 24150027

[31] Alexander EK, Pearce EN, Brent GA (2017). 2017 guidelines of the American Thyroid Association for the diagnosis and Management of Thyroid Disease during Pregnancy and the postpartum. Thyroid.

[32] Tranquilli AL, Brown MA, Zeeman GG, Dekker G, Sibai BM (2013). The definition of severe and early-onset preeclampsia. Statements from the International Society for the Study of hypertension in pregnancy (ISSHP). Pregnancy Hypertens.

[33] Steegers EA, von Dadelszen P, Duvekot JJ, Pijnenborg R (2010). Pre-eclampsia. Lancet.

[34] Zhang Y, Dai X, Yang S (2017). Maternal low thyroxin levels are associated with adverse pregnancy outcomes in a Chinese population. PLoS One.

[35] Klein I, Ojamaa K (2001). Thyroid hormone and the cardiovascular system. N Engl J Med.

[36] Turunen S, Vaarasmaki M, Mannisto T (2019). Pregnancy and perinatal outcome among hypothyroid mothers: a population-based cohort study. Thyroid.

[37] Korevaar TIM, Medici M, Visser TJ, Peeters RP (2017). Thyroid disease in pregnancy: new insights in diagnosis and clinical management. Nat Rev Endocrinol.

[38] Rotondi M, Cappelli C, Pirali B (2008). The effect of pregnancy on subsequent relapse from Graves' disease after a successful course of antithyroid drug therapy. J Clin Endocrinol Metab.

[39] Hernandez M, Lopez C, Soldevila B (2018). Impact of TSH during the first trimester of pregnancy on obstetric and foetal complications: usefulness of 2.5 mIU/L cut-off value. Clin Endocrinol (Oxf).

[40] Ong GS, Hadlow NC, Brown SJ, Lim EM, Walsh JP (2014). Does the thyroid-stimulating hormone measured concurrently with first trimester biochemical screening tests predict adverse pregnancy outcomes occurring after 20 weeks gestation?. J Clin Endocrinol Metab.

[41] Procopciuc LM, Caracostea G, Hazi G, Nemeti G, Stamatian F (2017). D2-Thr92Ala, thyroid hormone levels and biochemical hypothyroidism in preeclampsia. Gynecol Endocrinol.

[42] Gong X, Liu A, Li Y (2019). The impact of isolated maternal hypothyroxinemia during the first and second trimester of gestation on pregnancy outcomes: an intervention and prospective cohort study in China. J Endocrinol Investig.

